# Collaborative Learning Based Sybil Attack Detection in Vehicular AD-HOC Networks (VANETS)

**DOI:** 10.3390/s22186934

**Published:** 2022-09-13

**Authors:** Sofia Azam, Maryum Bibi, Rabia Riaz, Sanam Shahla Rizvi, Se Jin Kwon

**Affiliations:** 1 Department of Computer Science and IT, University of Azad Jammu and Kashmir, Muzaffarabad 13100, CO, Pakistan; 2 Raptor Interactive (Pty) Ltd., Eco Boulevard, Witch Hazel Ave, Centurion 0157, South Africa; 3 Department of AI Software, Kangwon National University, Samcheok 25913, Korea

**Keywords:** VANET, sybil attack, vehicular ad hoc network, machine learning

## Abstract

Vehicular Ad-hoc network (VANET) is an imminent technology having both exciting prospects and substantial challenges, especially in terms of security. Due to its distributed network and frequently changing topology, it is extremely prone to security attacks. The researchers have proposed different strategies for detecting various forms of network attacks. However, VANET is still exposed to several attacks, specifically Sybil attack. Sybil Attack is one of the most challenging attacks in VANETS, which forge false identities in the network to undermine communication between network nodes. This attack highly impacts transportation safety services and may create traffic congestion. In this regard, a novel collaborative framework based on majority voting is proposed to detect the Sybil attack in the network. The framework works by ensembling individual classifiers, i.e., K-Nearest Neighbor, Naïve Bayes, Decision Tree, SVM, and Logistic Regression in a parallel manner. The Majority Voting (Hard and Soft) mechanism is adopted for a final prediction. A comparison is made between Majority Voting Hard and soft to choose the best approach. With the proposed approach, 95% accuracy is achieved. The proposed framework is also evaluated using the Receiver operating characteristics curve (ROC-curve).

## 1. Introduction

VANET is a variant of Mobile Ad-hoc Network (MANET) that allows information to be shared between nearby automobiles and roadside equipment. The noteworthy features of VANET compared to MANET is the high mobility of vehicles and its dynamic nature i.e., it has no fixed network topology [1]. A VANET system comprises VANET-equipped automobiles with On-Board Units (OBUs) for network connection and communications, Road Side Units (RSUs), placed alongside roads for communication with the OBUs, and servers connected to the RSUs called trusted authority (TA) for collection and monitoring of the OBUs [2]. The highly mobile vehicles have wireless connection among vehicles and roadside-installed infrastructure devices.

The VANET’s vehicles are highly efficient, and they can store and interpret large data. WAVE protocol is used for communication in VANET [3]. According to the subsequent scenarios of VANETs, connectivity can be viewed as vehicle-to-vehicle (V2V) communications or vehicle-to-infrastructure (V2I) communications in VANETS [4]. Every vehicle in VANET has a traffic-related message to share with other vehicles in the network or with RSU within a specific time interval. The message contains information related to vehicle states (ID, location, speed, etc.) and its safety. Since VANET vehicles are free to travel in any direction, the pattern of their wireless communication with other vehicles and devices always changes [5]. When vehicles move in harsh environments exchanging information in real-time, sensors’ signal quality is badly affected by the dynamic environment. It can result in network attacks such as eavesdroppers, replay back attacks, Impersonation attacks, False attribute encompassing, Tunneling attacks, Message altering, ID disclosure, and the Sybil attack [6].

**Figure 1:**
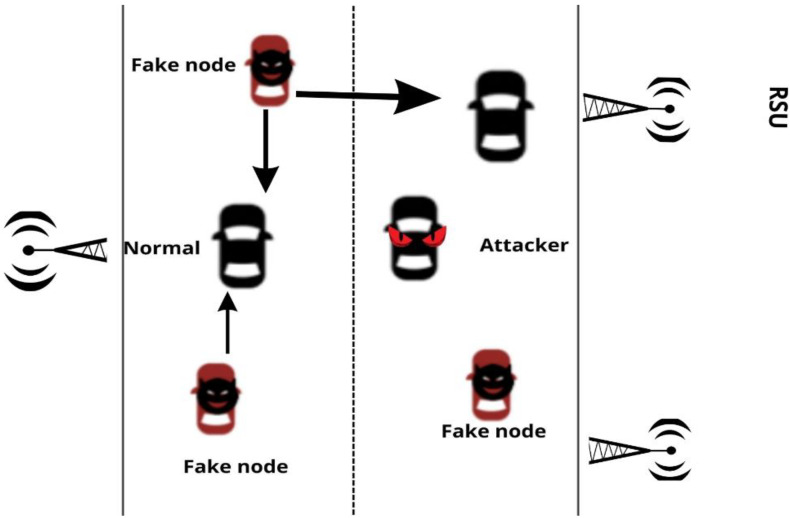
Sybil Attack scenario in VANET.

A Sybil attack involves an attacker node fabricating several fake identities and creating a fake traffic congestion scenario [7]. These nodes claim that there are more cars on the road. This scenario is shown in Figure 1 below. To disseminate false information across VANETs, a Sybil attacker will create many bogus identities. Sybil attacks can destabilize the normal operations of essential Intelligent Transportation System (ITS) applications, resulting in serious effects, including the cost of human life. For example, a malicious node may misleadingly report a traffic jam or accident to cause a nearby vehicle driver to reroute, potentially causing a real traffic jam on another road. Alternatively, the malicious node may exploit the fake virtual nodes to use a large amount of bandwidth, disrupting the dependable communication between Sybil nodes.

The Sybil attack, in particular, might seriously jeopardize key services and protocols that rely on the majority voting system [8]. Many privacy-preserving techniques including Public Key Infrastructure (PKI) and encryption techniques were observed, but still, VANETS is exposed to the Sybil attack.

The majority of known Sybil attack solutions are largely concerned with identifying the malicious node the adversary has created. In a Sybil attack, an adversary uses several identities to damage the reputation of an established system. False information propagated by a single vehicle may not be convincing. A given piece of information may need to be reinforced by several vehicles before the application accepts it as true. When a malicious node can impersonate numerous nodes and reinforce fake data, the other nodes in the system might believe this false information and make decisions based on it [9]. In this way, it can gain inappropriate power over the network by creating multiple fake identities. As a result, detecting and preventing Sybil attacks on VANETs become more critical. The majority of available methods for Sybil attacks detection use Received Signal Strength Indication (RSSI) to estimate the location of neighbor nodes and compare it to the geographic location claimed in the information received in the beacon message. Several approaches, on the other hand, are based on an information-theoretic context.

Summing up the discussion, in today’s technological age, cyberspace with all of its advantages and disadvantages is becoming a more important source of node-to-node information transfer [10]. VANETS have significantly developed as a result of recent advancements in wireless technology and the Internet of Things (IoT). Multiple studies on VANET and IoT demonstrate that both have significant impacts on smart transportation systems [11]. Though IoT is the new appearing technology and produces big data and its features bring convenience to us, threats also emerge with them [12]. VANETS, due to its dynamic nature and fast mobility behavior is always at risk of several security attacks among which the Sybil attack is the most appalling. Although privacy-preserving methods were observed for the detection of Sybil attacks, we need more robust and sophisticated security measures, that can learn from past experiences and identify both known and unidentified attacks. Machine learning algorithms are one of the most advanced tools for detecting cyberattacks [10].

Machine learning is playing a vital role in combatting a number of security attacks. Machine learning offers a wide range of applications in all fields of life including healthcare, business, agriculture, industries, and network security [13,14,15,16]. It has been extensively exploited in the field of cybersecurity in the last few decades [17,18]. It has shown remarkable performance in various fields for anomaly detection [19]. Machine learning has also gained the attention of ITS researchers in the last few decades. Considering the significance of Machine learning in the ITS field, this study also focuses on the detection of Sybil attack using Machine learning techniques. Following are the main contributions of this study.

- This study aims to detect Sybil’s attack in VANET using a collaborative machine learning technique based on Majority Voting.
- The proposed mechanism employs occasional features to describe a mobility pattern of vehicular nodes, allowing an evaluation of the mobility of real vehicle nodes in front of the inaccurate Sybil node displacement patterns.
- Different Sybil’s and non-Sybil’s scenarios are simulated in VANETS using SUMO and Ns2 for three different vehicle densities to evaluate the performance of the proposed technique in different environments. The data gathered through simulation are used to feed machine learning models.
- Supervised machine learning classifiers including K-Nearest Neighbor (K-NN), Naïve Bayes (NB), Decision Tree (DT), Support Vector Machine (SVM), and Logistic Regression (LR) are used as base classifiers in the proposed mechanism and are applied to the dataset in a parallel fashion.
- Majority voting mechanism based on Hard and Soft is adopted to predict the final output of classifiers
- A comparison between soft and hard majority voting is also presented at the end of the study.
- The results indicate that the proposed mechanism is a suitable strategy for detecting Sybil attacks and preserving VANET service delivery.

To the best of our knowledge, such a framework has not been investigated earlier. The rest of the paper is organized as follows: Section 2 presents previous contributions related to Sybil attack detection and a comparison of all mentioned machine learning techniques in comparison with the proposed technique. Section 3 entails system design, presenting a view of how simulation is done and how the Sybil attack is launched in the VANET system for the proposed study. Section 4 describes the proposed framework of the study; Section 5 presents the outcomes of the study including a comparison of both used techniques and individual classifiers used in a study with the same classifiers used in the previous studies.

## 2. Related Work

This section is divided into two parts. The first part of this section focuses on the threat to validity where database and search strings for organization and literature of study are discussed. The second and final part of this section covers significant research on VANET security as well as strategies against Sybil attacks. The previous studies that have been mentioned are highlighted in a table at the end of this section, along with how they differ from our strategy and their merits and demerits.

Threat to validity: We explored the following studies for this article (1) Any of the five stated base algorithms for Sybil attack detection in VANET, (2) Identifying Sybil attacks in VANETS using ensemble classifiers, (3) PCA, SMOTE, and the effects of combining PCA and SMOTE, (4) Discuss performance measures in terms of recall, accuracy, precision, and ROC.

The phrases such as “Sybil attack detection and machine learning techniques”, “Sybil attack detection in VANET using machine learning techniques”, “Sybil attack detection in VANET using ensemble classifiers”, and “machine learning and Sybil attack in VANETS” have all been used in different combinations to find peer-reviewed journal articles, conference proceedings, book chapters, and reports. We focused on the five databases: ACM Digital Library, IEEE Xplore, Science Direct, Springer Link, and Web of Science. Additionally, Google Scholar was used. Recent development over the past five to seven years had been our main focus. In total, 1952 papers were retrieved, and 1364 duplicate items were eliminated. In order to find potential papers, the title and abstract were evaluated.

A total of 113 papers’ whole texts were examined to determine their relevance to the inclusion criteria. In the next step, articles that discussed (1) the Sybil attack in networks other than VANETS and (2) Sybil attack detection methods other than Machine Learning were omitted. In addition to the specified criteria, forward and backward searches turned up 22 more studies. In total, 44 studies were finally selected for data extraction purposes.

To detect the Sybil attack in VANET, Zhou. T et. al proposed P2DAP; Privacy-preserving detection of Sybil attack in Vehicular Ad hoc Network [20]. P2DAP uses the hash value to provide different pseudonyms to the same vehicle. RSU received a single event from two pseudonyms with the same hash value; consider it as a Sybil node. This solution failed for the high-density vehicular network due to excessive communication between the nodes. Another method based on encrypted digital signals is proposed by Reddy in [21]. This method is based on creating a trust factor among vehicles by using digital signatures as a hash function. However, this method does not account for the nodes’ mobility behavior, which limits identification in high-mobility circumstances. In [22], similarity between the driving pattern of vehicles is measured using Mahalanobis Distance to detect the Sybil node. Later in the same year, a strategy for identifying Sybil attacks is developed using K-NN methods to categorize the nodes and distinguish Sybil nodes from other nodes [23].

Hamed et al. designed the infrastructure-based affinity computation (IOAC) method in [24]. This method observed the differences in movement changes among vehicles passing by RSU. He assumed that two or more vehicles passing multiple RSU at the same interval, having two distinct identities in the area of two different RSU is regarded as Sybil. However, the proposed method has scalability and efficiency issues. With the development of the IoT and the electronic sector, the localization of mobile anchor nodes is receiving a lot of attention. A range-free localization approach is proposed for sensors in a flying anchor-based three-dimensional (3D) wireless sensor network [25]. This setup is very similar to a vehicle-to-infrastructure (V2I)-based positioning technique. Using a distance matrix, this technique can help to determine the position of a node which can be very useful for the detection of an anomaly in VANET attacks.

Ensemble methods are also popular among the research community to identify the Sybil nodes. Bayesian classifier and Neural network in combination with deep learning are used in [26]. This approach showed a high detection rate in real-time using accuracy, F1-score, and sensitivity. However, the mobility pattern of the vehicle node is not considered. ADAS sensors are installed in modern vehicles that collect the information regarding the surrounding area including distance and angle from the vehicle [27]. This information is processed using a deep learning algorithm to detect the Sybil node. Extreme Learning Machine-based Sybil attack detection called SyDVELM is proposed in [28].

In this research, the attack is detected on the basis of information received by RSU as a Beacon message which contains necessary information regarding vehicle mobility patterns. SyDVELM is an artificial intelligence-based technique that uses ELM with occasional features of VANET mobility. The results show maximum detection rate, maximum scalability, and minimized accuracy time. Sybil node detection is also performed by S. Beg et al. using vehicle motion trajectories in [29]. This technique used a digital signature issued by RSU to determine the vehicle’s trajectory.

Helmi. Z et al. detect Sybil attack using deep learning [30]. By combining variables, this study was able to identify Sybil attack trends in one VANET area. The study used layer-by-layer protection (three layers) to predict accuracy of 94 percent. A behavioral-based classification was performed on the VeReMi dataset using ML classifiers, i.e., AdaBoost, Decision tree, and XGBoost and trained them using Eigenvalues [31]. A plausibility factor was added to improve the performance of the classifier. However, this study has shortcomings in the computation of Eigenvalues. In [32], Sybil attack is detected using classification algorithms SVM, Logistic regression, and Random Forest. The simulated VANET data are balanced using SMOTE and are fed to machine learning algorithms. This study showed remarkable accuracy performance but has less vehicle density as compared to the rest of the studies. A CMEHA-DNN-CMEHA-DNN-based intrusion detection system (IDS) centered on deep learning is proposed by [33] for identifying the Sybil attack in VANET. This method showed better results as compared to all existing methods. Table 1 summarizes the Machine learning studies that have been presented, highlighting how they differ from our approach as well as their benefits and drawbacks.

All these methods focused on calculating RSSI and comparing it with other factors to detect Sybil node, or they used neighbor vehicles to detect the Sybil vehicle based on several assumptions. However, when there are a lot of vehicle nodes, some methods that rely on an information-theoretic framework cannot scale. Additionally, the above-mentioned methods focused on either a single Machine learning classifier or a combination of two classifiers to detect Sybil attack. Some studies used more than two classifiers for attack detection, however, these classifiers were not used as ensemble or collaborative techniques. Ensemble methods are believed to be more accurate and robust as compared to individual classifiers and are the main focus of this study.

To the best of our knowledge, no study except [32] has attempted to make machine learning-based Sybil attack detection on a balanced VANET dataset. Ref. [32] applied SMOTE to the simulated VANET dataset and reports a decrease in classifier performance. Therefore, this research work aims to (1) apply balancing technique i.e., SMOTE to simulation dataset for different VANET scenarios, and (2) apply multiple machine learning algorithms used earlier for Sybil attack detection as base classifiers to ensemble majority voting technique.

## 3. System Design

In VANET, vehicles/nodes broadcast a message when they enter a network. This message is known as a beacon message [8]. This message is received by all other vehicles that fall in range and also received by RSUs installed on roadsides. Beacon messages contain the vehicle’s ID, address, position (x, y, z axis), speed, direction, event, and other information.

In the event of any emergency or disaster, such as road blockades, landslides, accidents, traffic jams, construction sites, and so on, a related beacon message might be used to distribute information and alert vehicles [34]. These safety messages can also be used to extract the information required by researchers and network administrators to take necessary decisions and actions in case of any security attack. In this research work, beacon messages are used for the detection of Sybil nodes.

The VANET Scenario for the study is created by simulating a vehicle network using two simulation tools, SUMO [35] and Ns2 [36]. By simulating various traffic patterns in the VANET, the data needed for machine learning are produced. There are two phases to VANET simulation. Urban mobility and road traffic simulation are included in the first section and are implemented using SUMO. The network communication is performed in the second phase and is carried out using Ns2.

### 3.1. Simulation Setup and Attack Model

In a vehicular communication context, Sybil attacks can be divided into 2 steps: virtual node generation and launch attack. According to ETSI standards, cooperative awareness messages (CAMs) could be used in the virtual node creation process and make virtual nodes recognizable to other ITS stations [26]. In terms of attack strategy, Sybil nodes can be considered of as rational and irrational attackers [37].

Irrational attackers do not desire a certain outcome and rational attackers have a specific aim. Only rational attackers are considered in this study since they are more hazardous and predictable.

SUMO is used to simulate automobile movements along roadways, at intersections, and in an urban environment. Simulation is performed on 300–800 vehicles in two lanes, 4 × 4 Manhattan grid with nine RSUs installed on the roadside. The vehicles move at a constant speed and have a communication range of 300 m. Three different VANET simulation scenarios were implemented with 300, 500 and 800 nodes, respectively. The number of Sybil nodes is also increased as we increase the number of normal nodes in simulation from 300 to 800. The attacker nodes attack in the high-density area at different time intervals, each node creating 15–20 fake identities. The simulation run for 300 s and constraints are established on real-time Urban Traffic. The resulting trace file is imported into Ns2 to simulate the communication setup. Static nodes are designed and set as RSU in Ns2 for receiving messages from vehicles. The simulation constraints are specified in Table 2.

### 3.2. Traffic Data

Dataset required for Machine learning models is generated using simulation tools in two steps. In the first step, Sumo is used to generate VANET traffic scenario in an urban environment. In the second step, Ns2 is used for network communication. Data packets contain important features such as sender ID, position, RSSI values, sender speed, acceleration, claimed position, claimed speed, GPS position, receiver id, and Receiver location. These data are stored in a base station and will be processed in the next section for defining the movement pattern of vehicles. Only important features needed for the creation of movement pattern is selected and will be fed to machine learning classifiers. Random vehicle states selected from simulation data are shown in Table 3.

## 4. Proposed Ensemble Framework for Sybil Node Detection

**Figure 2:**
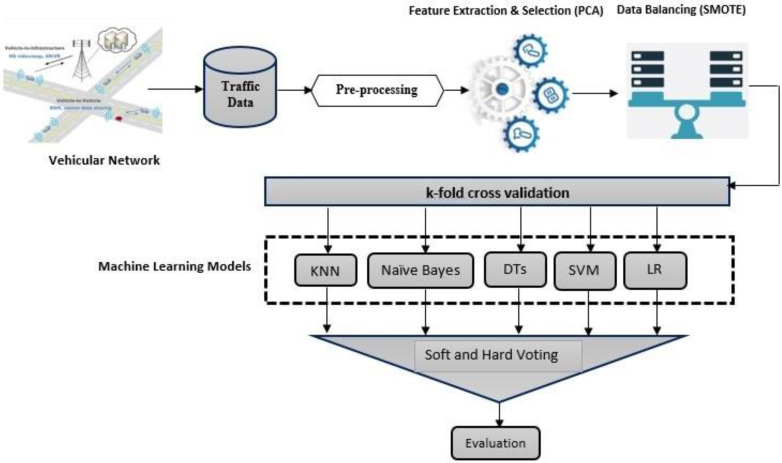
Proposed Framework for Sybil attack detection.

The proposed ensemble framework based on the Majority Voting method for the detection of Sybil attack is shown in Figure 2. Different classifiers including k-Nearest Neighbor (K-NN), Naïve Bayes, Decision Trees, SVM, and Logistic Regression are used in a parallel ensemble manner. The output from each classifier is then input to Hard and Soft majority voting mechanisms. The proposed framework works on the driving pattern of vehicle movement. This pattern contains the important features of simulated VANET nodes that help in determining the mobility pattern of vehicles. The proposed ensemble machine learning method is applied to this data, to detect the Sybil attack. The steps of the proposed framework are discussed in detail below.

### 4.1. Data Pre-Processing

The data generated in the previous section are now ready for pre-processing. Moreover, data transformations including scaling and standardization are performed [38]. Data labeling is also performed in this step. The useful features are selected and irrelevant ones are discarded. The final data contain important features such as time stamp, vehicle position (x and y; z is set to 0), vehicle speed including its direction acceleration, and acceleration difference. Features are selected based on the driving pattern of the vehicle. As we assume that there is more similarity in the driving pattern of vehicles when the density of the vehicle is high. From the data, we create a driving pattern *Mi* for every vehicle *Vi* in the network for each time interval t. The M of vehicle *V* for time interval *t* consists of 5 vectors. V⃗_x,1_ = (x _x,1_, x _x,2,_ x _x,3_, x _x,4_, x _x,5_) where;

- x _x,1_ represents vehicle time
- x _x,2_ represents vehicle location
- x _x,3_ represents vehicle speed
- x _x,4_ represents vehicle acceleration
- X _x,5_ represents acceleration difference

The driving pattern matrix *M_i_* for each vehicle *V_i_* for time interval *t_i_* in network defined by following values (for i = 1 − n).

$$M_{i}=\left[\begin{array}{ccccc} x_{11} & x_{12} & x_{13} & x_{14} & x_{15}\\ x_{21} & x_{22} & x_{23} & x_{24} & x_{25}\\ x_{31} & x_{32} & x_{33} & x_{34} & x_{35}\\ . & . & . & . & .\\ . & . & . & . & .\\ . & . & . & . & .\\ x_{n1} & x_{n2} & x_{n3} & x_{n4} & x_{n5} \end{array}\right]$$

This matrix represents the VANET’s movement patterns or an overall view of environmental behavior. In the next step, dimensionality reduction is performed for mitigating overfitting problems, reducing the computational cost, and for better visualization of data. For this purpose, Principle Component Analysis (PCA) is applied to the dataset [39]. It is an unbiased linearization method for extracting features and lowering dimensionality. PCA can be done either by Eigen Vector Decomposition (EVD) or by Single Value Decomposition (SVD). SVD is a generalization of EVD where we compute the singular value of the data matrix instead of the covariance matrix of data. The difference between the two is that EVD works well with symmetric and square matrices whereas SVD works well with non-square matrices. To reduce n × d movement pattern M to k dimensions where (k < d) PCA SVD is used. The following steps are involved in PCA:

1. Centralized the data
2. Calculate covariance matrix
3. Calculate the eigenvectors of the covariance matrix
4. Select m eigenvalues corresponding to m eigenvalues

The overall process of the PCA is the same for EVD and SVD. Only in steps 2 and 3 does it vary a little. In this research, we need to reduce data into two principal components so we set the n-component value equal to 2. To reduce n × d matrix M to k dimensions where (k < d) PCA SVD is used. The explicit explanation of SVD is given below:

For every matrix, M there exists factorization such that:

$$M_{mxn}=U_{nxn}\;\Sigma_{mxn}\;V_{mxm}^{T}$$

Here, U and V^T^ are orthogonal and Σ is a diagonal matrix and its elements are called singular values. They are non-negative numbers and in descending order of their magnitude (σ_1_ ≥ σ_2_ ≥ σ_3_ … σ_p_ ≥ 0). Now, for covariance matric R we have;

(1) $$\;\;R=\frac{M^{T}M}{n\;-1}$$

Solving (1)

(2) $$R=\frac{V\Sigma^{T}U^{T}\cdot \;U\Sigma^{T}V^{T}}{n\;-1}$$

Here, U^T^ and U are orthogonal so U^T^U = I. Now we have:

(3) $$R=\frac{V\;\Sigma^{T}\Sigma \;V^{T}}{n\;-1}$$

So Σ^T^Σ = Σ^2^ and Σ^2^ is a diagonal matrix where diagonal elements are λ (Eigenvalues) and are eigenvalues of covariance matrix where λ^i^ = σ^2^/n − 1 and V are Eigenvectors of the covariance matrix. Principal components can be obtained with MV = UΣ.

Balancing a dataset is a necessary step before applying a machine learning algorithm. The data are considered to be imbalanced if classifying groups are not equally distributed. Real-world datasets frequently contain a large percentage of “normal” cases (majority group) and a very small number of “abnormal” or “interesting” examples (minority group). Additionally, indeed the expense of incorrectly classifying a minority group (interesting) sample as a normal example is frequently higher than the expense of the opposite error. For balancing the dataset, a popular SMOTE sampling technique [40] is examined. The number of malicious nodes will be less than the number of benign nodes when taking into account the real traffic scenario.

As a result, there was an inequity in the amount of examples for malicious and non-malicious nodes. SMOTE is used to address the issue of unbalanced classes. SMOTE duplicates the under-sampled data to provide balanced data. Data balancing technique is usually performed before feature extraction (PCA), but applying SMOTE right after PCA improves the accuracy [39].

### 4.2. Sybil Attack Detection

In this segment, the proposed Ensemble Majority voting technique to identify Sybil attack is presented in detail. Initially, the data from the vehicle nodes is obtained, as discussed in Section 3.2. The data is then analyzed to obtain important data on the movement pattern of the mobile nodes, and the matrix is represented by two high-energy eigenvalues. As a result of this process, the understudied classifiers receive these two most appropriate eigenvalues as input. The VANET’s nodes’ eigenvalues are utilized to build a classification model.

#### 4.2.1. K-Fold Cross-Validation

Cross-Validation refers to a resampling mechanism that generates data (training and test) samples in order to evaluate the machine learning models. In this method, the number of k sets (folds) are extracted for sample placement, hence named k-fold cross-validation [41]. k-fold cross-validation splits the whole data into k mutually exclusive folds based on random policy. In general, each fold is of approximately equal size. On these data folds, training and testing are then repeated k times. One fold is utilized as the test set in an iteration, while all other folds are used as training data to create the trained model. In this paper, the collected and sampled data are divided into training and test using 10-fold cross Validation i.e., k = 10.

#### 4.2.2. K-Nearest Neighbor(K-NN)

The K-Nearest Neighbor (K-NN) aims to compute the similarity of a new data point to the categories/classes [41]. The parameter k represents the number of nearest (similar) neighbors. The K-NN algorithm will be experimented multiple times with different values of k to identify the k with minimum error rate while maintaining the system’s ability to make good predictions on VANET simulation data. Different values of k are examined. After testing the differed values to choose the correct value of k, it is observed that KNN gives stable predictions on k = 5.

#### 4.2.3. Naïve Bayes

Bayesian classification aims to infer a class label for instances with unknown class labels using probability estimation [41]. It calculates membership probabilities for each class, such as how likely it is that a certain one belongs to that class. The Naïve Bayes follows the mathematical relation specified below:

(4) p(H|X)=p(X|H)p(H)/p(X) 

where *p*(*H*|*X*) shows the posterior probability for a hypothesis/class *H*. It is dependent upon instance *X*. The posterior probability for *X* given *H* is represented by *p*(*X*|*H*). Additionally, *p*(*H*) denotes the prior probability for (*H*). Meanwhile, for example, *p*(*X*) displays previous probability (*X*). For all classes, *p*(*X*) remains constant. Therefore, to determine its maximum value for a specific class, only *p*(*H*|*X*) needs to be computed.

#### 4.2.4. Decision Tree

A decision tree is a supervised learning algorithm (classifier). A non-parametric supervised learning technique that may be applied to both classification and regression is decision trees. By learning fundamental decision rules from data attributes, the objective is to build a model that forecasts the value of a target variable [41]. The decision tree classifier builds a classifier by creating a tree structure to achieve the goal. The nodes in the decision tree represent a test for a given attribute. While the possible output is represented by the edge descending from the node. The process is repeated recursively on every subtree until the final class label is found.

#### 4.2.5. Support Vector Machine (SVM)

Support vector machine is a classification algorithm. It classifies the instances into relevant categories/classes. It initially transforms the original data into higher dimensionality using nonlinear mapping. It then searches for a decision boundary that isolates the instances of one class from another. The decision boundary is the optimal linear hyperplane [41]. The SVM classifier identifies this hyperplane with the help of support vectors and margins. The margin shows the maximum separation between categories/classes. A separating hyperplane can be written as *w·x + b =* 0 for separating the hyperplane for a two-class classifier. In this relation, x represents training instances, w and b show weight vector and bias, respectively. In this research, ThunderSVM is investigated with rbf kernel.

#### 4.2.6. Logistic Regression

It is a predictive analytical technique based on probabilities. Similar to a linear regression model, a logistic regression model uses a cost function that is more complicated and is known as the “sigmoid function” or the “logistic function” [34]. When developing neural networks for deep learning, the concept of logistic regression as a building block can be helpful.

#### 4.2.7. Majority Voting (Hard and Soft)

Majority Voting is a mechanism that computes the cumulative majority of votes for each output class, rather than building separate specialized models and determining their performance. There are two methods for obtaining a majority vote: hard voting and soft voting.

**Figure 3:**
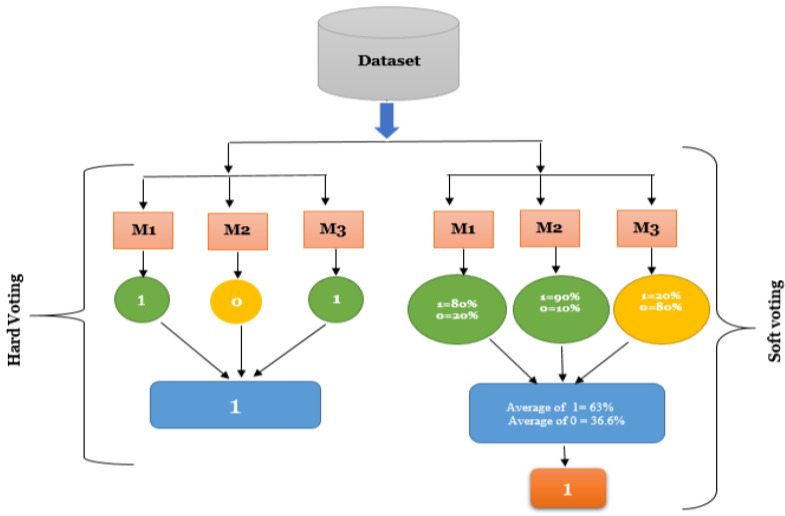
Majority Voting Methods.

We choose both soft and hard majority voting for our study. The hard majority voting works by assigning the class label predicted by majority classifiers to the instance as shown in Figure 3. It can be seen that if three classifiers assign a label of “1” while one classifier assigns a label of “0” then, according to the hard majority voting principle, the final label will be assigned as “1”.

Soft majority voting considers results of all individual classifiers, averages their results and predict the final result. It works by assigning a probability value to each individual classifier that a given data point belongs to a specific target class as shown in Figure 3. The results are aggregated and assigned weights based on the classifier’s relevance. The target label with the greatest sum of weighted probabilities receives the maximum votes. In the soft voting technique, the final target label is predicted based on the greatest aggregate of weighted probabilities.

#### 4.2.8. Evaluations

The performance measures such as accuracy, recall, precision, and area under the curve are used for evaluation.

The Accuracy describes the model’s performance across all classes. It is the ratio of number of correct predictions to the total no of predictions. In our case, it is the number of correctly classified Sybil samples to the total number of samples [17].

(5) $$\mathrm{Accuracy}=\frac{\mathrm{Number}\;\mathrm{of}\;\mathrm{correct}\;\mathrm{predictions}}{\mathrm{Total}\;\mathrm{number}\;\mathrm{of}\;\mathrm{predictions}}$$

Recall specifies the ratio of the total number of relevant samples to the correct positive outcomes. In our case, it is the total number of Sybil samples to correct classified samples. Recall reflects how well the model can distinguish Positive samples [17].

(6) $$\mathrm{Recall}=\frac{\mathrm{True}\;\mathrm{positive}}{\mathrm{True}\;\mathrm{positive}+\mathrm{False}\;\mathrm{Negative}}$$

Precision shows the quantity of correct positive outcomes divided by the classifier’s predicted quantity of positive outcomes. In our case, it is the ratio of correctly classified Sybil samples to all classified Sybil samples in the dataset. A greater precision number is preferable and reflects better classifier performance [17].

(7) $$\mathrm{Precision}=\frac{\mathrm{True}\;\mathrm{positive}}{\mathrm{True}\;\mathrm{positive}+\mathrm{False}\;\mathrm{Positive}}$$

The ROC [42] curve is drawn which depicts the measurement of sensitivity by drawing a function with (1-specificity). Generally, its value lies between 0 and 1, the closer the value to 1, the better the performance will be. It is the most frequently used graph, that plots the values of TPR (y-axis) versus FPR (x-axis) and gives a summary of the classifiers’ performance of all thresholds [17]

## 5. Results and Discussion

In this section, the experimental results of the proposed framework are reported. Experiments are conducted on three different Node sizes i.e., 300, 500, 800.

Evaluation is performed in three dimensions:

- Performance evaluation of proposed ensemble framework using classifiers based on Majority Voting (Hard and Soft)
- Comparative analysis between soft majority voting and hard majority voting methods
- Comparative analysis between the proposed framework and earlier investigated methods used for Sybil node detection

### 5.1. Performance Evaluation of Majority Voting

The proposed framework investigated using ensemble majority voting for detection of Sybil attack in VANET as explained in Section 4. Considering both types of Majority Voting (Hard and Soft) evaluation matrices are calculated for each dataset as shown in Table 4.

It can be observed from Table 4 that as the dataset increases, some of the performance metrics for the individual classifiers decrease. A crucial aspect of classifying Sybil nodes is fewer packets for fake identities which affect the performance measures [32]. It can also be observed that some individual classifiers such as K-NN and Decision tree show better or equal performance to Ensemble Majority Voting (Soft). This is because the Individual base algorithms can be improved with the help of an ensemble. However, when introduced to classifiers with very poor performance compared to the rest, the performance of ensemble classifiers can drop over [43]. Keeping our focus on the proposed technique, it was observed that both classifiers (Hard and Soft) show the same performance for small vehicle datasets (300 and 500). However, when examining the large dataset (800) vehicles, Majority Voting Soft shows higher performance as compared to Majority Voting Hard but equal or better performance as compared to some individual classifiers.

### 5.2. Effect on Applying SMOTE on Dataset

The proposed methodology used SMOTE for balancing the dataset before applying Machine learning classifiers. For the dataset of 300 and 500 nodes, the imbalance dataset show better performance as compared to balanced data using SMOTE. However, for 800 nodes, an increase in performance is seen for all evaluation metrics used in study. So assuming the real-world urban scenario, where there is a dense vehicle environment, the proposed approach is believed to perform better.

**Figure 4:**
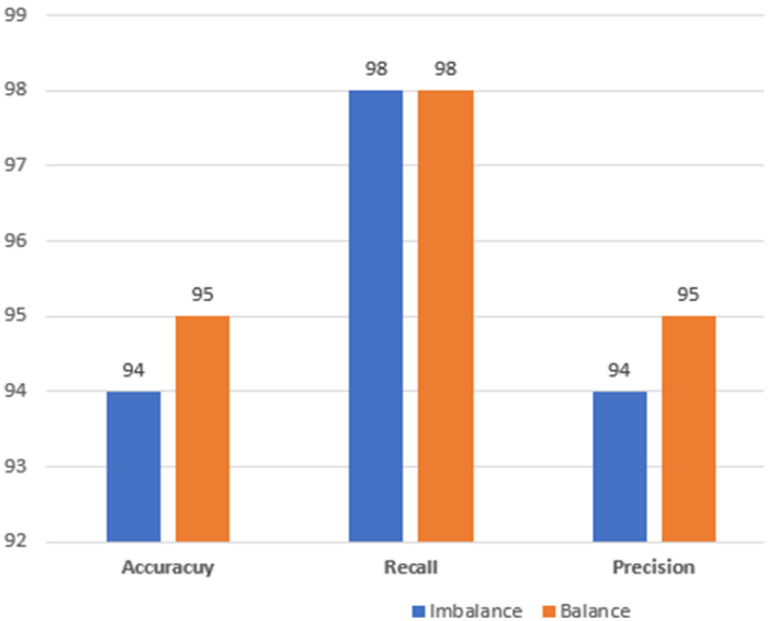
Performance Evaluation for Balanced and Imbalanced Data.

A bar chart showing the performance of imbalance and balance data is shown in Figure 4 below.

### 5.3. Comparative Analysis between Majority Voting Hard and Soft

**Figure 5:**
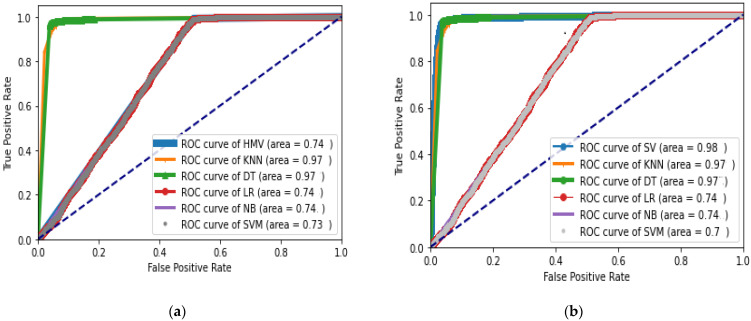
ROC curve Showing performance of (**a**) Majority Voting Hard and (**b**) Majority Voting Soft.

**Figure 6:**
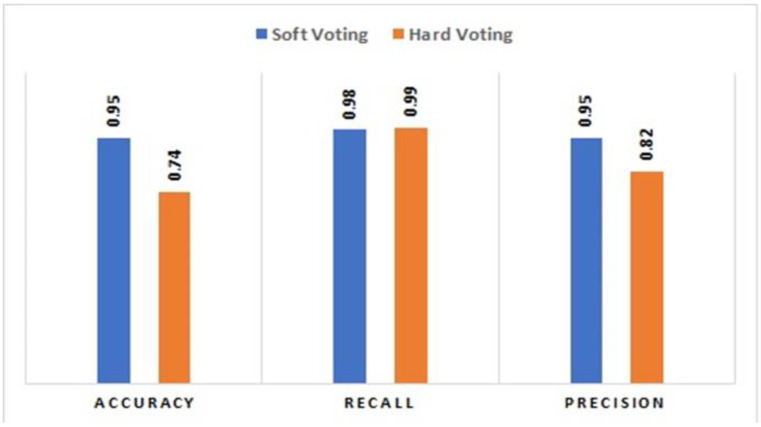
Bar Chart showing performance of Majority Voting Hard and Soft for (800 Nodes data).

A comparative analysis between Majority voting Hard and Soft is made using the dataset of 300, 500 and 800 nodes. During experimentation, it was observed that the results of the evaluation metrics for both Majority Voting Hard and Soft are the same for datasets of 300 and 500. However, for the data of 800 nodes, Majority Voting Soft is showing better performance as compared to Majority Voting Hard for the mentioned observation that the performance of both classifiers varies for 800 nodes data. ROC graphs are drawn for both classifiers. It can be seen through the Figure 5 below that Soft Majority Voting (b) is showing better performance as compared to Hard Majority Voting (a). A bar chart is also shown in Figure 6, which specifies the performance of each evaluation matrix used in the study for both Hard and Soft voting for 800 vehicles datasets. From the chart, it can be seen that majority voting soft gives better performance on the 800 vehicles dataset as compared to Majority Voting Hard.

### 5.4. Comparative Analysis between the Proposed Ensemble Framework and Earlier Investigated Classifiers

A comparative analysis is performed between the classifiers used in previous studies K-NN [23], SVM [32] and logistic regression [32] with Majority Voting Soft using the proposed methodology.

***K-Nearest Neighbor (K-NN):***

- K-NN shows the same performance for the dataset of 300 and 500 nodes with accuracy, recall, and precision values of 99%, however Majority Voting soft shows a little decrease in performance as compared to KNN with accuracy, recall and precision values of 97, 98 and 97 percent, respectively.
- For the dataset of 800 nodes the K-NN shows 95% accuracy, 96% recall and 95% precision. Majority Voting Soft has equal performance to K-NN for the 800 nodes dataset. Majority Voting Soft shows accuracy and precision values of 95% same as K-NN, however recall value of Majority Voting Soft is higher as compared to KNN i.e., 98%.

***Support Vector Machine (SVM):***

- For the dataset of 300 nodes, SVM shows 89% accuracy, 83% recall and 90% precision. The performance of the proposed Majority Voting Soft is higher for the same data with accuracy, recall, and precision values of 97%, 98%, and 97%, respectively.
- The performance of SVM increases for the dataset of 500 nodes as compared to a dataset of 300 nodes. Now, accuracy and precision values for SVM are 96% and recall is 95%. In comparison to SVM, Majority Voting Soft has a slight increase in performance.
- For 800 nodes data, the performance of SVM decreases to 74 and 82 percent for accuracy and precision, respectively. Performance of SVM usually decreases on large datasets such as network and system data [44]. Majority Voting Soft for the same experiment show 95% accuracy and precision values. However, the recall value for both individual classifier (SVM) and ensemble classifier (Majority Voting Soft) is the same i.e., 98%.

***Logistic Regression:***

- For the dataset of 300 and 500 nodes, logistic regression shows accuracy and precision of 94%. However, the recall value of logistic regression is higher for 500 nodes as compared to the 300 nodes i.e., 98 percent. In comparison to the same data, the proposed Majority Voting Soft shows higher performance for both datasets.
- Logistic regression shows a high decrease in accuracy for data of 800 nodes in comparison to Majority Voting Soft. Logistic regression shows an accuracy of 74% whereas the accuracy value of Majority voting Soft is 95% which is reasonably high. The recall values for both classifiers are the same i.e., 98%. The precision of Majority Voting Soft is 95% which is greater as compared to the precision values of Logistic regression i.e., 82 percent.

## 6. Conclusions and Future Directions

VANET being a promising technology is constantly under threat of a number of the security attacks. These security attacks, specifically the ones focusing on security disruption and fake information dissemination i.e., Sybil attack, not only disturb the network flow but can be a severe danger to human life. Within this objective, this study proposed an Ensemble Majority Voting approach for the detection of Sybil attack.

The proposed ensemble method aims to have a more reliable prediction as compared to previous studies where a single classifier is used for attack detection. The proposed ensemble majority voting (Hard and Soft) is applied to the dataset acquired through simulation of real urban scenarios. The result shows that majority Voting Soft can better detect the Sybil attack with high accuracy as compared to Majority Voting Hard. In the future, we intend to test the proposed method using additional ensemble techniques, such as stacking.

## Figures and Tables

**Table 1:** Summary of previous Machine Learning work and proposed technique for Sybil attack detection.

Proposed ML Techniques	Advantages	Disadvantages	References
**SVM and KNN**	High detection frequency in a low-density environment.	Computational complexity is high.	[22,23]
**Deep Learning** **(In combination with Bayesian and Neural network)**	High detection rate in real-time.	Vehicle mobility patterns weren’t taken into account.	[26]
**Extreme** **Learning Machine** **(SyDVELM)**	High Performance and less time complexity.	No analysis in the low-density environment.	[28]
**Deep Learning** **(Neural Network architecture)**	Less time complexity.	Fewer variables were considered in the analysis. Results could be made better by including more variables.	[30]
**AdaBoost, XGBoost and DT**	Tested on the VeReMi dataset, shown good performance.	High time complexity	[31]
**SVM, Logistic regression and Random Forest**	High-performance rate	Less number of vehicles were used in simulation and attack strategy	[32]
**Collaborative Learning(Majority Voting)**	Simple and has High performance	Can be improved when tested with better resources and also with other ensemble techniques	

**Table 2:** Simulation Parameters.

Simulation	Constraints
Simulator	Sumo and Ns2
Time	300 s
Distance	1 km
Street Lane	Double Lane
Vehicle Speed	40–60 km/h
Total number of vehicles in simulation	300–800
Communication Range	300 m

**Table 3:** Datasheet showing some randomly chosen data points from simulation data.

Time	Location		Speed	Acceleration	Acceleration Difference
	X-axis	Y-axis			
0.753	365.804	289.84	0.4598	6.8844	6.8843
0.775	335.75	144.285	0.4513	9.0976	0.9706
0.794	780.624	232.654	0.4790	15.2455	14.7678
0.800	190.757	580.954	0.3977	4.9510	4.5510

**Table 4:** Results of proposed Majority Voting Technique (Hard and Soft).

Classifiers Used in study	300 Nodes Data			500 Nodes Data			800 Nodes Data		
	Accuracy	Recall	Precision	Accuracy	Recall	Precision	Accuracy	Recall	Precision
**Naïve Bayes**	0.85	0.98	0.88	0.83	0.98	0.86	0.71	0.99	0.81
**Decision Tree**	0.99	0.99	0.99	0.99	0.99	0.99	0.95	0.97	0.96
**K-NN**	0.99	0.99	0.99	0.99	0.99	0.99	0.95	0.96	0.95
**Logistic Regression**	0.94	0.96	0.94	0.94	0.98	0.94	0.74	0.98	0.82
**SVM**	0.89	0.83	0.90	0.96	0.97	0.96	0.74	0.98	0.82
**Hard voting**	0.97	0.98	0.97	0.97	0.98	0.97	0.74	0.99	0.82
**Soft voting**	0.97	0.98	0.97	0.97	0.98	0.97	0.95	0.98	0.95

## Data Availability

Not applicable.
